# Relapsed Brucellosis Manifested as Endocarditis with Vegetation on the Implantable Cardioverter Defibrillator Lead

**DOI:** 10.1155/2021/9925775

**Published:** 2021-06-30

**Authors:** Bisher Sawaf, Wael Kanjo, Yasir Alabbas, Ahmad Hatim, Unus K. Bedardeen, Wafer A. Dabdoob

**Affiliations:** ^1^Internal Medicine, Department of Medical Education, Hamad Medical Corporation, Doha, Qatar; ^2^Department of Cardiology, Heart Hospital, Hamad Medical Corporation, Doha, Qatar

## Abstract

Implantable cardioverter defibrillator lead endocarditis due to Brucella melitensis is a rare and life-threatening complication of brucellosis. Successful management requires a combination of medical treatment and device extraction. We present a case of relapsing brucellosis manifested as infective endocarditis colonizing the lead of the implantable cardioverter defibrillator with formation of vegetation on the lead. A 63-year-old male presented to the rehabilitation unit with hypotension. No other signs of infection were noted. The patient had a history of drinking unpasteurized milk since childhood and a previous episode of Brucella infective endocarditis. A transthoracic echocardiography showed an oscillating vegetation on the lead of the tip of the right atrial ICD, and the blood cultures were positive for Brucella melitensis. Surgical removal of the device was infeasible, and medical management was the only feasible option in this case.

## 1. Introduction

Brucellosis is one of the most encountered endemic zoonoses in many developing regions including the Middle East (ME), sub-Saharan Africa, South Asia, and South America [1]. This disease is caused by Brucella melitensis, a gram-negative, nonencapsulated, nonmotile, facultative intracellular coccobacillus [2, 3]. The mode of transmission of this infection is from infected animals to humans through either direct contact such as cut and aerosol inhalation or indirect contact such as ingestion of unpasteurized dairy products, urine, tissues, and products of conception (placenta) [4, 5]. Consumption of unpasteurized dairy products from contaminated milk is the main pathway of spread globally, especially in the Middle East [6].

Brucellosis presents with a broad spectrum of clinical manifestations including fever, arthralgia, myalgia, and back pain, and it is sometimes complicated with endocarditis, neurobrucellosis, and epididymo-orchitis [7]. Cardiovascular involvement is prevalent in 1% of brucellosis patients, with endocarditis being the most serious form and leading to brucellosis-related mortality [7, 8]. Although endocarditis caused by infection of a prosthetic device or implant is a rare complication of brucellosis, the most suitable therapeutic approach in this scenario is to remove the device. However, oftentimes the presence of multiple comorbidities and other factors, such as age, renders surgical management an infeasible approach, and the only option is to retain the device and place the patient on a prolonged antimicrobial treatment [9]. Due to the low number of reported cases of brucella infective endocarditis with device colonization, there is lack of consensus on the most appropriate approach for the management of these patients who are not candidates for surgery [10, 11].

A review of the literature revealed that about 25% of the reported cases of Cardiac Device-Related Brucella Endocarditis (CDRBrE) were in the Middle East [11–16]. In this report, we aim to describe the first case of recurrent brucellosis manifested as infective endocarditis colonizing the lead of the implanted cardioverter defibrillator (ICD) with formation of vegetation on the lead. To the best of our knowledge, this is also the first study to provide a comprehensive summary of CDRBrE cases reported in the literature.

## 2. Case Presentation

A 64-year-old Egyptian gentleman residing in Qatar, he has a long-standing history of hypertension, atrial fibrillation, and idiopathic dilated cardiomyopathy with a low ejection fraction (LV EF 26%), underwent implantation of a cardiac resynchronization therapy device (CRT-D) in 2013. Two years later, the CRT-D was downgraded to ICD.

On February 11th, 2020, the patient suffered from an ischemic stroke in the right middle cerebral artery for which he required decompressive craniectomy. Shortly after the procedure, his hospital stay was complicated with fever and malaise. Ingestion of partially boiled cow's milk was noted from the history. Blood cultures were positive for Streptococcus pneumoniae and Brucella melitensis. Based on these results, he was diagnosed with pneumonia and brucellosis, and antibiotics were commenced for treatment. As a result of the stroke, the patient suffered from hemiplegia which left him bed-bound and shifted to a rehabilitation centre for continuity of care. There, the patient showed only minor improvements in speech, but none in mobility. Nevertheless, the family refused to transfer him to a multispecialty hospital that provides medical, surgical, and long-term care.

**Figure 1:**
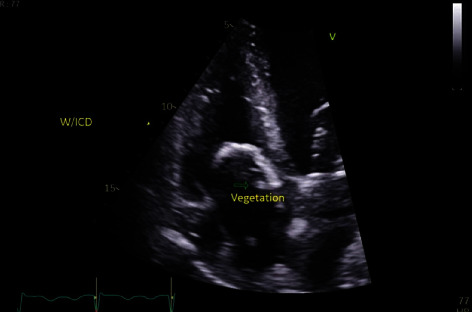
Vegetationson top of ICD lead. Transthoracic echocardiography, apical four-chamber view showing a large vegetation on the top of ICD atrial lead in a case of brucella endocarditis.

On November 20th, 2020, while the patient was still in the rehabilitation centre, his health started to deteriorate. He was found to be hypotensive with a blood pressure of 80/40, but afebrile with an oral temperature of 37.0°C. His heart rate and respiratory rate were 93 beats/minute and 18 breaths/minute, respectively. Physical examination revealed no skin changes over the ICD pocket, no cardiac murmurs on auscultation, and no apparent localized signs of infection. Blood tests revealed a normal white blood cell count (5 × 103 cells/mm^3^) and high C-reactive protein level (77 mg/L). Blood cultures were positive for Brucella melitensis. These tests were followed with transthoracic echocardiography which showed a single oscillating mass measuring 0.8 × 0.4 cm attached to the tip of the right atrial lead of the ICD suggestive of vegetation (Figure 1).

The patient was then transferred to the Heart Hospital as a case of relapsing brucellosis with brucella endocarditis and ICD-lead colonization. The Infectious Disease Team was consulted, and three-drug regimen was commenced including rifampicin (600 mg/day), gentamicin (5 mg/kg/day), and doxycycline (200 mg/day). Gentamicin was then increased to 7 mg/kg/day as the postdose peak levels were low, and trimethoprim/sulfamethoxazole (960 mg/12 h) was added to the treatment, with a recommendation to remove the ICD. A transesophageal echocardiography (TEE) was tried but was technically difficult to conduct due to the restricted neck movements and limited jaw mobility of the patient, which rendered the TEE probe impossible to insert. Therefore, a positron emission tomography (PET) scan was performed to confirm the nature of the vegetation, yet the results were being inconclusive.

The case was discussed in a multidisciplinary team (MDT) meeting, and all possibilities to deal with this infected ICD wires were explored. Due to the unavailability of laser-assisted lead extraction, surgery was proposed as an option; however, because of the patient's general condition, surgical removal was considered a high-risk procedure, and the final decision was to keep the ICD in place and to treat him conservatively with antibiotics. Another PET scan was requested that was also inconclusive, and a repeated echocardiogram revealed a slight increase in vegetative size (0.8 × 0.6 cm). Eventually, Gentamicin was stopped due to renal impairment.

On December 17th, 2020, the patient was medically fit for discharge and was sent home with oral antibiotics for 6 weeks.

On March 29^th^, 2021, the patient was doing well and still on oral antibiotics.

## 3. Discussion

Brucella infective endocarditis is a rare and life-threatening complication of brucellosis. A systematic review assessing the clinical manifestations of brucellosis revealed brucellosis-associated endocarditis was prevalent in an overall 1% of patients [7]. Another study found that 1 in 23 CDRIE cases was caused by Brucella melitensis [15]. Nevertheless, despite its low prevalence and insidious progression, infective endocarditis is a very severe complication of brucellosis, and it has been reported in a few of Cardiac Device-Related Infective Endocarditis (CDRIE) cases. In our review of 14 published CDRBrE cases, we found that 11 patients were males, two were females, and one was unidentified, none of them with involvement of the device lead. Moreover, their ages ranged between 38 and 82 years old (Table 1).

Diagnosing brucellosis begins with an assessment of epidemiological factors. Our patient mentioned a history of drinking unpasteurized milk since childhood. A history of contact with animals (sheep and goats), consumption of dairy products, or consumption of uncooked meat was noted in several studies as a common trait of the majority of brucellosis patients [11, 12, 16–19]. An assay of blood, bone marrow, or other body fluids or tissue cultures are considered the gold standards for the diagnosis of brucellosis.

However, it is imperative to underscore that Brucella is a fastidious bacteria requires special culture media with a variable and more prolonged incubation period, which could justify why only seven patients had positive blood cultures (Table 1). There are multiple serological tests used to diagnose brucellosis, with the most common being the serum agglutination test. In general, agglutination titres ≥ 1 : 160 are considered diagnostic, although a titre of ≥1 : 320 is considered diagnostic in endemic areas. Enzyme-linked immunosorbent assay (ELISA) is another popular serological method used to diagnose brucellosis. ELISA detects total Brucella immune-specific IgM, IgA, and IgG immunoglobulins [20–22].

Optimal management of CDRIE should include removal of the whole device and prolonged antibiotic therapy to eradicate the pathogen from the body, especially in the presence of unusual organisms such as Brucella melitensis. This treatment is also recommended when CDRIE is suspected in the case of occult infections without any known source of disease [9]. Considering the inherent risk of an open surgical operation, our patient was deemed a high-risk candidate for surgical device removal and medical management was adopted in place [23]. The same approach was reported in two other studies on brucella CDRIE with good outcomes. In both studies, the patients had an implanted pacemaker which was not removed, while they received medical treatment for brucellosis. Follow-up examination revealed that they were both in good condition without any remaining signs of infection, and no recurrence of the illness was reported (Table 1) [10, 11]. On the other hand, other studies show that medical therapy alone is associated with high risk of disease recurrence and mortality [24]. It remains to be seen in the near future whether our patient and the patients in the previous studies will remain disease-free [10, 11]. In most cases of brucella CDRIE, device removal and lead extraction followed by extended antibiotic therapy are required for the effective treatment of this disease (Table 1).

Nuclear studies such as positron emission tomography (PET) scan and single photon emission computed tomography (SPECT) are used in the diagnosis of the difficult cases of device-related endocarditis [25]; however, in our patient, the result of PET scan was inconclusive because of the intense physiologic left ventricular myocardial uptake, and the area of the right ventricle lead could not be evaluated.

The proposed therapy for brucellosis endocarditis by the European Society of Cardiology (ESC) includes doxycycline (200 mg/24 h), cotrimoxazole (trimethoprim and sulfamethoxazole) (960 mg/12 h), and rifampin (300–600/24 h) for 3–6 months, with some recommending gentamicin to be added for the first 3 weeks, while the addition of streptomycin (15 mg/kg/24 h in 2 doses) for the first few weeks is optional [9]. An alternative regimen includes doxycycline and rifampin for a total of 6 weeks, but this regimen shows a higher failure rate, and it is not recommended for osteoarticular disease [25]. The relapse rate in patients treated with the latter is 14.4% and 22.6% [26, 27]. In our case, we followed the recommendations of ESC and used a combination of rifampicin (600 mg/day), doxycycline (200 mg/day), septrin (trimethoprim and sulfamethoxazole) (960 mg/12 h), and gentamicin (7 mg/kg/day); the latter administered at a low dose for fear of renal impairment. Only one study adopted this medical management and reported good outcomes with no relapse up to date [11]. A study that reported multiple relapses used rifampicin (600 mg/day) and minocycline (100 mg bd), for 12 weeks; rifampicin (600 mg/day) and ciprofloxacin (500 mg bd), for 6 weeks; rifampicin (600 mg/day), trimethoprim-sulfamethoxazole (2 g intravenous bd), and minocycline (100 mg bd); rifampicin, ciprofloxacin, and minocycline, for 6 weeks, on 4 separate occasions for treatment [10].

Interestingly, brucella CDRIE can occur practically at any time after device implantation, where relapse of brucella CDRIE was noted in six cases as well as in our patient (Table 1) [10, 13, 16, 17, 28, 29]. Routine follow-ups should be a requirement in cases of systemic brucella infection to avoid overlooking the rare complication of relapsing infective endocarditis. This must be implemented in the care plan of our patient and any future patients when surgical intervention is impractical.

## 4. Conclusion

The present case report highlights the possibility of brucella being an agent that can complicate the placement of an ICD. A high degree of awareness of the possibility of colonization of implanted devices with brucella in patients residing in endemic regions is fundamental for early laboratory recognition of this virulent pathogen to ameliorate the course of the disease. Routine follow-ups must be conducted on patients with ICD recovering from brucellosis who are not good candidates for surgical management.

## Figures and Tables

**Table 1:** Case reports of brucellosis involving pacemakers and cardiac devices.

Study/year	Country	Gender	Age (years)	Type of device	Brucella relapse	Brucella blood culture	Miscellaneous cultures	Device and leads removed	Antibiotics	Treatment duration
de la Fuente et al. [28]	Spain	Male	63	Chamber end cavitary pacemaker	Yes	No	Pacemaker, leads, pus, necrotic tissue	Yes	Doxycycline Rifampin Streptomycin	45 days 45 days 21 days
Francia et al. [29]	Spain	Male	71	Pacemaker	Yes	No	Pacemaker and leads. Negative TEE	Yes	Doxycycline Rifampin	6 weeks 8 weeks
Ulkar et al. [12]	Turkey	Male	68	Pacemaker	No	Yes	Abscess culture	Yes	Doxycycline Rifampin	6 weeks 6 weeks
Miragliotta et al. [10]	Italy	Female	70	Single-chamber pacemaker	Yes	Yes	Cutaneous swab from fresh wound of recent removal and replacement of pacemaker	No	Rifampicin Ciprofloxacin Minocycline	6 weeks 6 weeks 6 weeks
Dourakis et al. [17]	Greece	Male	70	ICD	Yes	Yes	TEE showed mass on tricuspid, pacemaker, and leads had vegetation	Yes	Doxycycline Ciprofloxacin Rifampin	12 months 12 months 12 months
Al-Majid [13]	Saudi Arabia	Male	38	Pacemaker	Yes	Yes	Leads	Yes	Doxycycline Rifampin	6 weeks 6 weeks
Gungor et al. [14]	Turkey	Male	61	Pacemaker	No	Yes	Ultrasonography revealed an abscess and a fistula. Cultures from sputum samples, drained materials from the abscess, and the pacemaker pocket	Yes	Doxycycline Rifampin	6 weeks 6 weeks
Osmonov et al. [15]	Turkey	N/A	N/A	Pacemaker	N/A	Yes	Lead culture TEE showed vegetation	Yes	N/A	6 weeks
Elmardi et al. [16]	Saudi Arabia	Female	54	ICD	No	No	TEE showed large masses attached to all leads. Culture from pocket fluid and tissues	Yes	N/A	N/A
	Saudi Arabia	Male	52	Single-chamber ICD	Yes	No	Culture from pocket tissue	Yes	Cotrimoxazole Doxycycline Gentamicin	N/A
Al-Adsani and Al-Mousa [11]	Kuwaiti	Male	70	Pacemaker	No	Yes	Transthoracic echo showed calcification and thickening of the aortic valve with two mobile masses attached to the aortic valve. TEE was unremarkable.	No	Doxycycline Rifampin Cotrimoxazole Gentamicin	3 months 3 months 3 months 2 weeks
Dhand and Ross [18]	United States	Male	66	ICD	No	No	Cultures from superficial specimen, tissue from the pocket, ICD, and leads	Yes	Gentamicin Ciprofloxacin Doxycycline	6 weeks 6 weeks
Enjalbert et al. [30]	France	Male	65	ICD	N/A	No	Culture from device	Yes	N/A	N/A
Kanji and Saxinger [19]	Canada	Male	82	Pacemaker	No	No	Cultures from leads and ventricular tissue	Yes	Gentamicin Doxycycline Rifampin	12 weeks 12 weeks

## Data Availability

The data used to support the findings of this case study are stored in the patient file in the Cerner but restricted by the HMC ethics board in order to protect patient privacy. Data are approachable by the corresponding author and other researchers who meet the criteria for access to confidential data.

## Abbreviations

ME: Middle East
CDRIE: Cardiac Device-Related Infective Endocarditis
ICD: Implantation of cardioverter defibrillator
CRT-D: Cardiac resynchronization therapy device
TEE: Transesophageal echocardiography
PET: Positron emission tomography
ESC: European Society of Cardiology.
